# Stronger Affinity of Water-Soluble Cationic Porphyrins to Cancer DNA under Acidic Conditions as a Basis for Selectivity

**DOI:** 10.1021/acsomega.6c01892

**Published:** 2026-07-01

**Authors:** Nelli H. Karapetyan, Artem V. Badasyan, Gayane V. Ananyan

**Affiliations:** † Department of Physics, 105430 Yerevan State University, Al. Manoogian 1, 0025 Yerevan, Armenia; ‡ Materials Research Laboratory, University of Nova Gorica, Vipavska 13, SI-5000 Nova Gorica, Slovenia

## Abstract

The affinities of porphyrins to healthy and cancer DNA under physiological and acidic conditions were studied. Titration of *meso*-tetra­(4-*N*-hydroxyethylpyridyl)­porphyrin and its Ag-substituted derivative solutions with DNAs, isolated from healthy and tumor tissues, has been performed. Using the simple neighbor-exclusion model of McGhee and von Hippel with the binding isotherm formula optimized for fitting errors, we found the binding constant *K*_b_ and exclusion parameter *n* from the titration absorption spectra. It was shown that the binding parameters of porphyrins to DNA under acidic conditions are higher than those under physiological conditions. The *K*_b_ of porphyrins with cancer DNA at pH 6.4 is an order of magnitude higher, and *n* is lower than that of healthy DNA at pH 7.4. The enhanced affinity of porphyrins for cancer DNA indicates that conformational alterations in the DNA structure create more-accessible binding sites. This selective interaction underscores the potential of porphyrins as effective agents for a targeted anticancer therapy.

## Graphical abstract

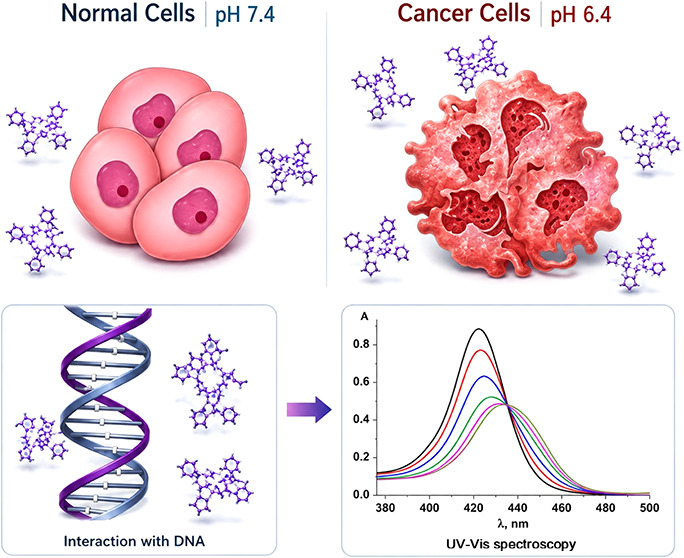

## Introduction

Porphyrin-type compounds play an essential role in nature. Green plant pigments, such as chlorophylls, contain magnesium and are crucial for photosynthesis. Heme, a red blood pigment containing iron, is another important metal-containing porphyrin. Heme is part of hemoglobin, carrying oxygen, and cytochromes, facilitating metabolic processes. Myoglobin, a heme-containing protein, supports tissue respiration and oxygen saturation in the muscle tissue. Cobalamin, or vitamin B12, containing cobalt, acts as a carrier of groups in biosynthesis, and its deficiency causes severe anemic diseases.^1^ Both natural and synthetic porphyrins are important organic chromophores that are used in various fields because of their unique properties. In biomedicine, they are employed as biosensors for cancer imaging and in photodynamic therapy. Porphyrins interact with cellular macromolecules, such as proteins, DNA, and RNA. Some porphyrin compounds are used as radiosensitizers in cancer therapy, targeting cellular DNA.^2^ Porphyrins are particularly attractive candidates for cancer therapy because of their ability to preferentially accumulate in cancer cells and interact with cancer-associated DNA structures. After localizing in the nucleus and mitochondria, photoactivated porphyrins generate reactive species that induce DNA damage, leading to selective apoptotic or necrotic death of malignant cells.^3,4^ Furthermore, certain porphyrins exhibit a strong affinity for guanine-rich G-quadruplex DNA regions, which are frequently associated with oncogenes and cancer cell proliferation, making them important therapeutic targets.^5^ Upon light activation, porphyrin binding can promote site-specific DNA strand cleavage at these regions, enhancing the selective destruction of cancer cells while minimizing the effects on healthy tissues.^6,7^ These same porphyrins, when administered to animals, preferentially accumulate in cancerous tissues while being rapidly cleared from normal tissues.^8^

Acid–base balance (pH) is a critical factor for cellular viability and function. It has been reported that glucose metabolism becomes altered during the transformation of normal cells into cancer cells. As a result of dysregulated glycolytic metabolism, the extracellular pH of solid tumors becomes acidic because of the high rates of lactic acid production and accumulation.^9^ Environmental acidity promotes tumor progression by causing genomic instability and facilitating local invasion and metastasis. Tumor cells consume large amounts of glucose to generate ATP and support proliferation and, even in the presence of oxygen, produce lactate, which contributes to acidification of the tumor microenvironment. Under normal conditions, blood lactate concentrations range from 0.5 to 2.5 mmol/L; however, during carcinogenesis, these levels may increase to 10–30 mmol/L. Consequently, physiological blood pH is maintained within a narrow range of 7.35–7.45, whereas the extracellular pH of the tumor microenvironment is often decreased to 6.0–6.5. This acidic milieu has been linked to enhanced metastasis, angiogenesis, therapeutic resistance, and increased tumor aggressiveness.^10–12^ Accordingly, pH 7.4 and pH 6.4 were selected as representative values for normal physiological conditions and the tumor microenvironment, respectively. As to *in vitro* studies of DNA–porphyrin interactions under acidic pH, the literature is very poor. The only related publication by Zhao et al.,^13^ reports increase of binding affinity of a cationic porphyrin toward G-quadruplex and duplex DNA at acidic pH. Based on these considerations, we sought to determine the effect of environmental acidity on the adsorption of the studied porphyrins on normal and cancer DNA. Our previous *in vivo* studies demonstrated the anticancer activity of *meso*-tetra­(4-*N*-hydroxyethylpyridyl)­porphyrins (H_2_TOEPyP4 and AgTOEPyP4).^14^ The aim of the present study is to compare the interactions of H_2_TOEPyP4 and AgTOEPyP4 with DNA isolated from healthy and cancerous tissues under physiological and acidic pH conditions *in vitro*. Specifically, we aim: (i) to evaluate the thermodynamic favorability of the interactions between the studied porphyrins and cancer DNA; (ii) to compare these interactions with those observed for healthy DNA; and (iii) to assess the role of an acidic environment in porphyrin–DNA binding.

The present study is limited to *in vitro* models. Investigations using cancer cell lines will be planned for future work.

## Materials and Methods

All reagents and chemicals used in this study were of analytical grade and were obtained from Sigma-Aldrich (St. Louis, MO, USA). The water-soluble cationic porphyrins H_2_TOEPyP4 and AgTOEPyP4 were synthesized at the Department of Pharmaceutical Chemistry, Yerevan State Medical University.^15^ The molecular masses of H_2_TOEPyP4 and AgTOEPyP4 are 940 and 1152 Da, respectively. The chemical structures of the studied porphyrins are shown in Figure 1.

**Figure 1:**
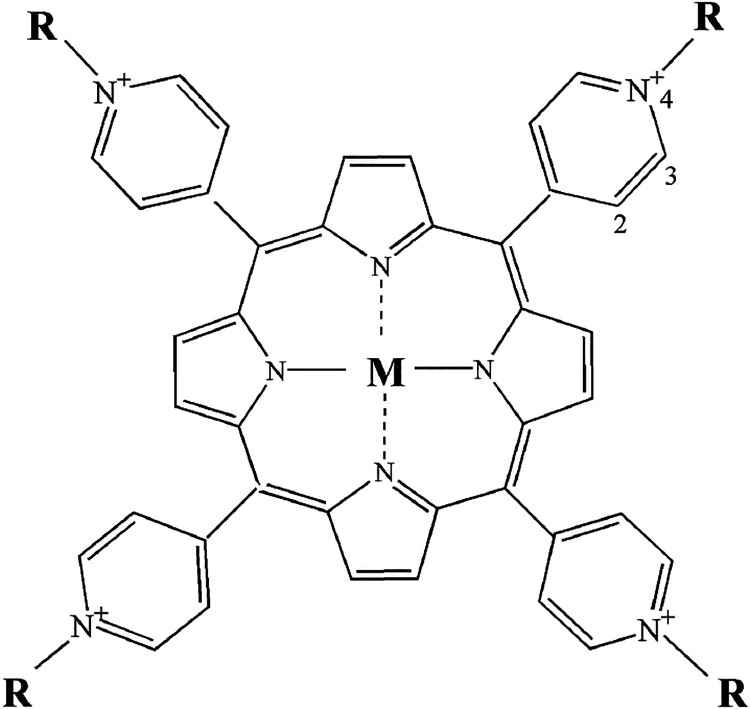
Chemical structure of H_2_TOEPyP4 and AgTOEPyP4 porphyrins. M = 2H, Ag; R = CH_2_–CH_2_–OH.

Male white Wistar rats weighing 120–140 g were used in the experimental studies. The animals were divided into two groups of 10 rats each. Samples collected from each group were divided into five portions for independent DNA extraction. All data are presented as the mean of five independent measurements with standard deviation. Group I consisted of healthy animals and served as the control group. In Group II, carcinoma was induced by local administration of a single 15 mg dose of 7,12-dimethylbenz­[*a*]­anthracene (DMBA). The treatment protocol is described in detail in ref 14.

DNA was isolated from healthy rat liver tissue and rat carcinoma tissue using a standard chloroform extraction method.^16^ Following each extraction, the DNA purity was assessed spectrophotometrically. Absorbance measurements at 230, 260, and 280 nm were used to evaluate the sample purity. The DNA samples exhibited *A*_260_/*A*_280_ and *A*_260_/*A*_230_ ratios of 1.8 and 2.0, respectively, indicating the absence of significant contamination.

The interactions of H_2_TOEPyP4 and AgTOEPyP4 with cancer DNA were studied under physiological and acidic conditions and compared with their interactions with healthy DNA. Stock solutions of the porphyrins were prepared in distilled water at a concentration of 10^–3^ M. DNA concentrations were determined using the molar extinction coefficient ε_260_ = 1.31 × 10^4^ M^–1^ cm^–1^ and were expressed in base-pair equivalents. The concentration of the DNA stock solution was 0.3 × 10^–3^ M (bp), whereas the porphyrin concentration was maintained at 10^–6^ M in all experiments. Titration was performed for each portion of isolated DNA, i.e., five replicates were performed. The Student’s *t* test was used to statistically evaluate the results, with *P* < 0.02 values considered statistically significant.

### Melting

DNA melting curves were recorded using a Lambda 800 UV/vis spectrometer (PerkinElmer). In all experiments, the heating rate was 0.5 °C/min, while the absorbance at 260 nm was continuously monitored. Measurements were performed over the temperature range of 25–95 °C using thermostated quartz cuvettes with a 10 mm optical path length.

### Microcalorimetry

Differential scanning microcalorimetry (DSC) of DNA samples was performed using a Nano-DSC differential adiabatic scanning microcalorimeter (TA Instruments). The volume of the sample cell was 0.6 mL, the heating rate was 0.5 °C/min, and measurements were carried out over the temperature range of 4–100 °C. The melting enthalpy (Δ*H*) was determined from the area under the heat-capacity curve according to the following relationship: Δ*H* = ∫*C*_p_d*T*, where *C*_p_ is the specific heat capacity at constant pressure and *T* is the temperature.

### Agarose Gel Electrophoresis

DNA samples (4 μg per lane) were analyzed by electrophoresis on 1% agarose gels using Tris–borate–EDTA buffer (pH 8.0) under condition of 5 V/cm. Electrophoresis was performed for 2.5 h. Following electrophoresis, the gels were stained with ethidium bromide (0.5 μg/mL), visualized under a UV transilluminator, and photographed. A 1 kbp (kilobase pair) DNA Ladder (Promega, USA) was used as a molecular size marker to estimate the lengths of double-stranded DNA fragments in the range of 250–10,000 bp.

### Spectroscopy

All spectroscopic measurements were performed in biphosphate-buffered saline (BPSE) containing 0.6 mM Na_2_HPO_4_, 0.2 mM NaH_2_PO_4_, 0.185 mM NaCl, and 0.1 mM EDTA (ionic strength of 0.02) at pH 7.4 and 6.4. All reagents used were of high-purity analytical grade. The pH of the solutions was measured by using a Beckman pH meter (USA).

Absorption spectra were recorded at 25 °C in the visible region using a Lambda 800 UV/vis spectrophotometer (PerkinElmer). No porphyrin aggregation was detected at the relatively low concentrations used (10^–6^ M) under either pH 7.4 or pH 6.4 conditions. Titrations were performed by successive additions of a DNA stock solution to a 1 cm path length cuvette containing the porphyrin solution. The titration was continued until no further shift in the absorption maximum (λ_max_) of the free porphyrin was observed. The concentration of free porphyrin was determined from the experimentally measured absorption spectra as follows

1 $$C_{f}=C_{t}\cdot \frac{A-A_{\min}}{A_{\max}-A_{\min}}$$

where *A*_max_, *A*, and *A*_min_ are the absorbances of free, partially bound, and fully DNA-bound porphyrin, respectively: *C*_t_, *C*_f_, and *C*_b_ = *C*_t_ – *C*_f_ are the total, free, and bound porphyrin concentrations, respectively; *r* = *C*_b_/*C*_DNA_ is the binding ratio, defined as the concentration of bound porphyrin relative to the total DNA concentration; and *C*_DNA_ is the total DNA concentration.

In their seminal work, McGhee and von Hippel^17^ proposed a simple neighbor-exclusion model based on the assumption of an infinitely long linear DNA lattice containing identical, noninteracting ligand-binding sites. This model yields an equation that expresses the relationship between *r*/*C*_f_ and *r* as a function of the binding constant, *K*_b_, and the exclusion parameter, *n*, where *n* represents the number of DNA base pairs occupied by a single bound ligand and thereby rendered inaccessible to subsequent ligand binding

2a $$\frac{r}{C_{f}}=K_{b}\left(1-\mathrm{nr}\right){\left[\frac{1-\mathrm{nr}}{1-\left(n-1\right)r}\right]}^{n-1}$$

However, eq 2 is not suitable to process experimental results with the least-squares fitting. As noted by Correia and Chaires,^18^ nonlinear least-square analysis assumes that all experimental uncertainties reside in the dependent variable, which is not the case for eq 2: the quantity *r* is present on both left and right. Both *r* and *C*_f_ are subject to experimental error and the error in both of these quantities is propagated into the calculation of *r*/*C*_f_, a fact generally ignored. In order to avoid these problems, Correia and Chaires suggested to explicitly expressing the concentration of free porphyrins as a function of other model parameters^18^

2b $$C_{f}=r{\left(\frac{1-\mathrm{nr}}{1-\mathrm{nr}+r}\right)}^{-n}{\left[K_{b}\left(1-\mathrm{nr}+r\right)\right]}^{-1}$$

In this form, the McGhee–von Hippel model allows the binding constant (*K*_b_) and exclusion parameter (*n*) to be determined by nonlinear regression using eq 3. As shown later in this paper, this approach yields excellent fits with negligible fitting errors. All nonlinear regression analyses were performed using GraphPad Prism (GraphPad Software, San Diego, CA, USA).

## Results

We first characterized the structural features of normal and tumor DNA by using thermal denaturation, differential scanning microcalorimetry, and agarose gel electrophoresis.

### Melting of DNA

Figure 2 shows the differential melting curves corresponding to the helix–coil transition of normal and tumor DNA. The principal parameters derived from the melting curves are the melting temperature (*T*_m_), defined as the temperature at which 50% of the DNA base pairs are denatured; Δ*T*, the width of the melting interval; and Δ*h*, the DNA hypochromicity. Analysis of the melting curves revealed that tumor DNA exhibited a lower melting temperature and hypochromicity (*T*_m_ = 68.9 ± 0.1 °C; Δ*h* = 28 ± 0.1%) and a larger melting interval (Δ*T* = 9.7 ± 0.2 °C) than normal DNA (*T*_m_ = 71.5 ± 0.1 °C; Δ*h* = 35 ± 0.2%; Δ*T* = 7.1 ± 0.15 °C). These results indicate reduced structural stability of DNA isolated from tumor tissue. The decrease in hypochromicity suggests the presence of partially unwound regions in tumor DNA, whereas the broader melting interval indicates increased heterogeneity and defects in its secondary structure. The observed differences in the structure of normal and tumor DNA may also be caused by changes in the primary structure of tumor DNA, which are likely due to hypermethylation of cytosine in tumor DNA.^19,20^

**Figure 2:**
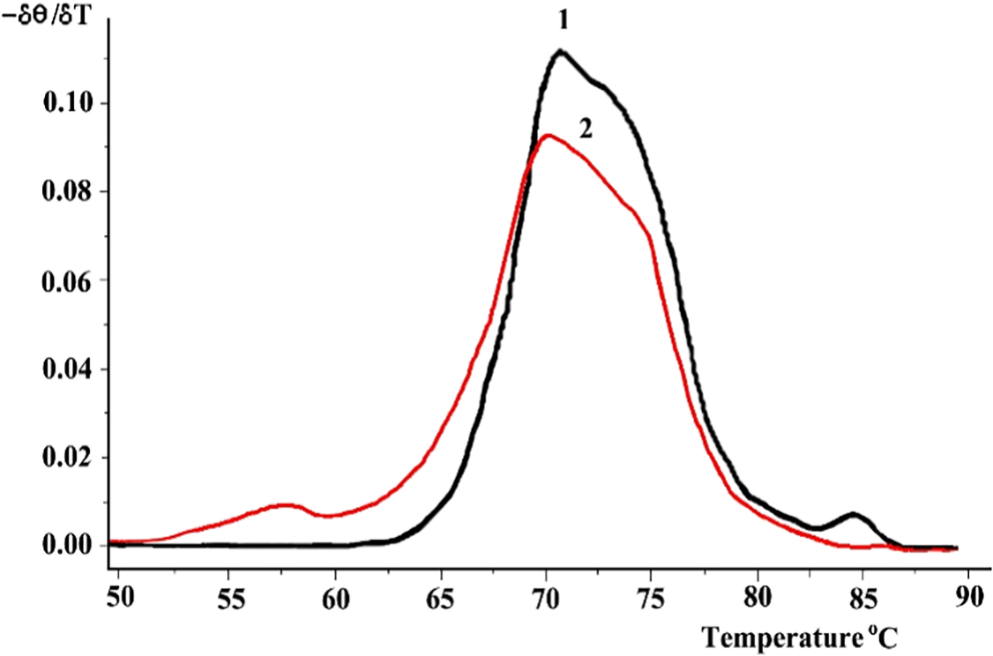
Differential melting curves of DNA isolated from healthy (1) and cancer (2) tissues of rats.

As shown in Figure 2, the differential melting curve of normal DNA exhibits a distinct melting peak at 84–86 °C, corresponding to a satellite CG-rich region, whereas this peak is absent in tumor DNA. In contrast, the differential melting curve of tumor DNA displays a thermolabile domain in the low-temperature region (55–60 °C), which is typically associated with the melting of AT-rich DNA regions. Because methylated cytosine can undergo spontaneous deamination to thymine, CG-rich regions may gradually be converted into AT-rich regions during DNA replication and repair processes. This change in base composition is consistent with the thermal behavior observed in tumor DNA.

Microcalorimetric analysis further revealed that the enthalpy of the helix–coil transition was substantially lower for tumor DNA (Δ*H* = 5.5 ± 0.03 kcal/mol) than for normal DNA (Δ*H* = 9.9 ± 0.05 kcal/mol). The reduced transition enthalpy of tumor DNA provides additional evidence for the presence of structural defects and decreased stability of the DNA double helix.

### Electrophoretic Analysis

Electrophoresis is a powerful molecular technique for distinguishing between normal- and tumor-derived DNA. As shown in Figure 3, the control sample (lane 2) exhibited a homogeneous band with a sharp boundary, which is characteristic of high-molecular-weight DNA. In contrast, DNA isolated from tumor tissue (lane 3) lacked the distinct high-molecular-weight fraction observed in healthy tissue. Whereas DNA from healthy tissue produced a narrow band centered near 15 kbp (estimated according to logarithmic extrapolation of the standard for DNA Ladder, lane 1), tumor DNA exhibited a substantially broader distribution ranging from approximately 12 to 6 kbp.

**Figure 3:**
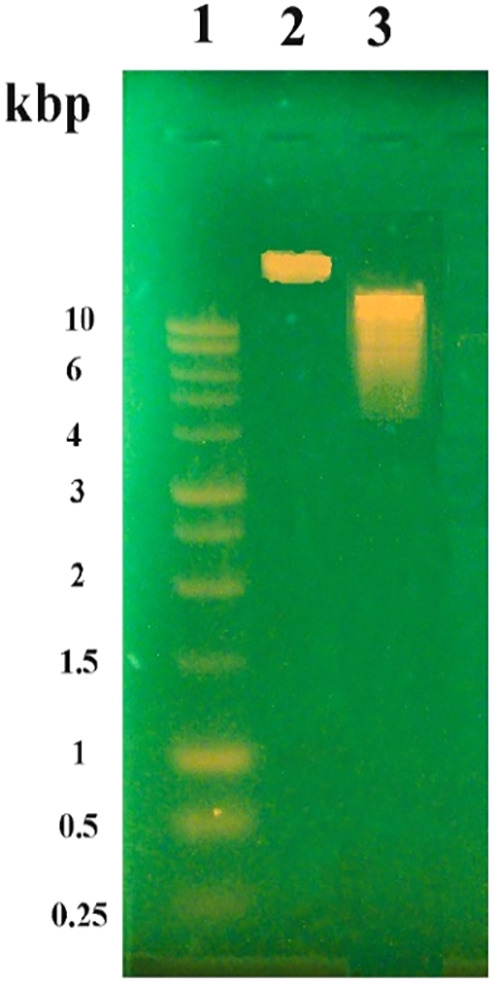
Agarose gel electrophoresis of DNA (4 μg per lane). Standard DNA Markers (1), healthy DNA (2), and cancer DNA (3).

The presence of DNA fractions with increased electrophoretic mobility is indicative of structural defects, including single- and double-strand breaks and fragmentation of tumor DNA.^21^ Thus, the electrophoretic results are in good agreement with the thermal denaturation and microcalorimetric data, both of which indicate a reduced structural integrity of tumor DNA.

### Absorption Spectroscopy

The results of the spectrophotometric titrations of H_2_TOEPyP4 and AgTOEPyP4 with DNA are presented in Figures 4 and 5. In all experiments, the porphyrin concentration was kept constant, while aliquots of the DNA stock solution were added gradually. Upon DNA addition, the Soret absorption band of both porphyrins shifted to longer wavelengths and exhibited a decrease in the absorbance intensity. Such hypochromic effects are commonly attributed to the formation of more ordered chromophore assemblies upon binding to double-helical DNA.^22^ As the DNA concentration increased, additional binding sites became available for porphyrin molecules, resulting in progressively greater hypochromicity until the binding equilibrium was reached. Titration was continued until further additions of DNA produced no detectable changes in the absorption spectra. In all cases, clear isosbestic points were observed, indicating the predominance of a single binding mode for both H_2_TOEPyP4 and AgTOEPyP4 under the experimental conditions.^23,24^

**Figure 4:**
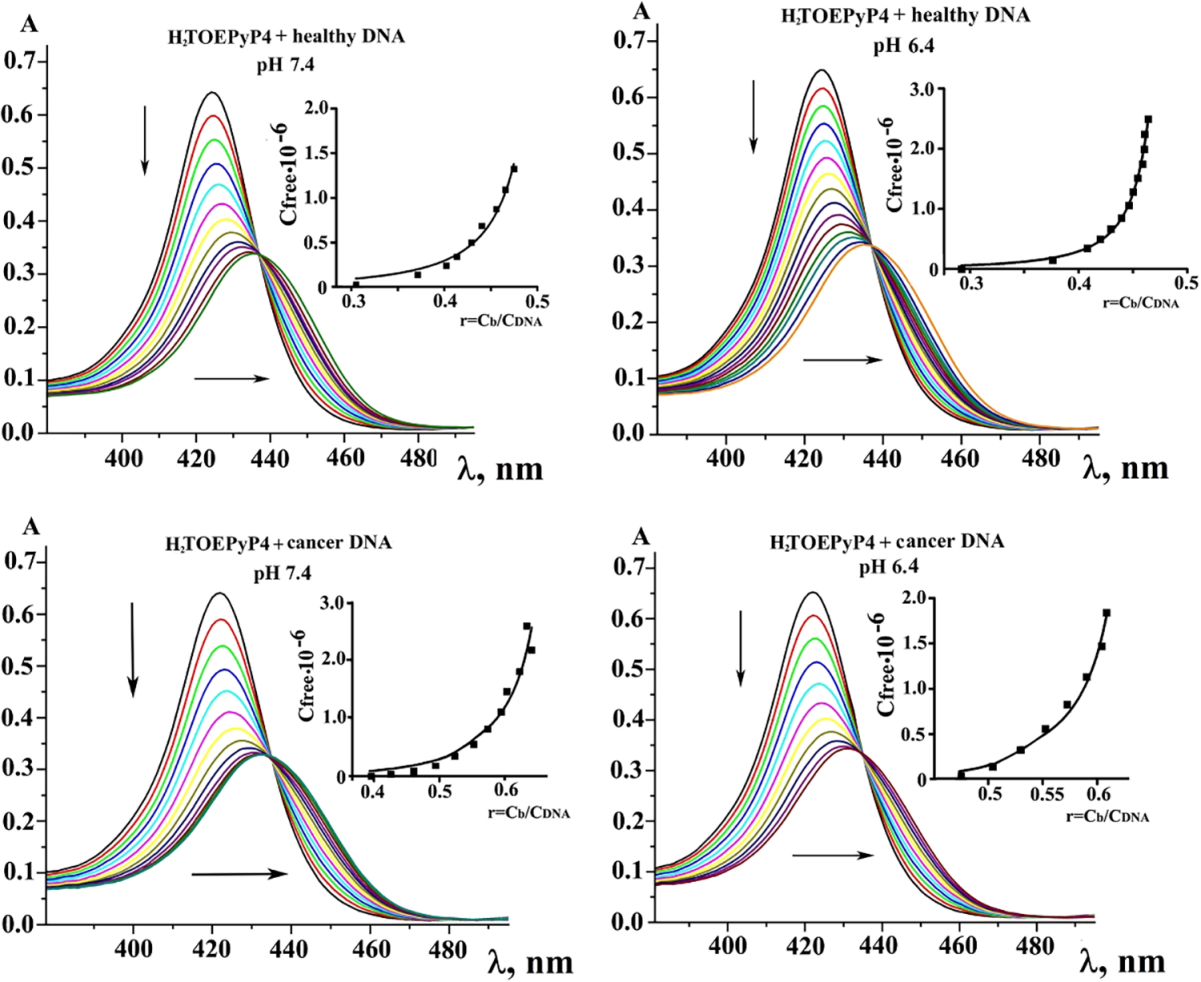
Absorption spectra and corresponding binding isotherms (insets) of H_2_TOEPyP4 in the Soret region obtained during titration with healthy and tumor DNA at pH 7.4 and pH 6.4. The insets show the results of fitting eq 3 to the experimental data processed according to eq 1.

**Figure 5:**
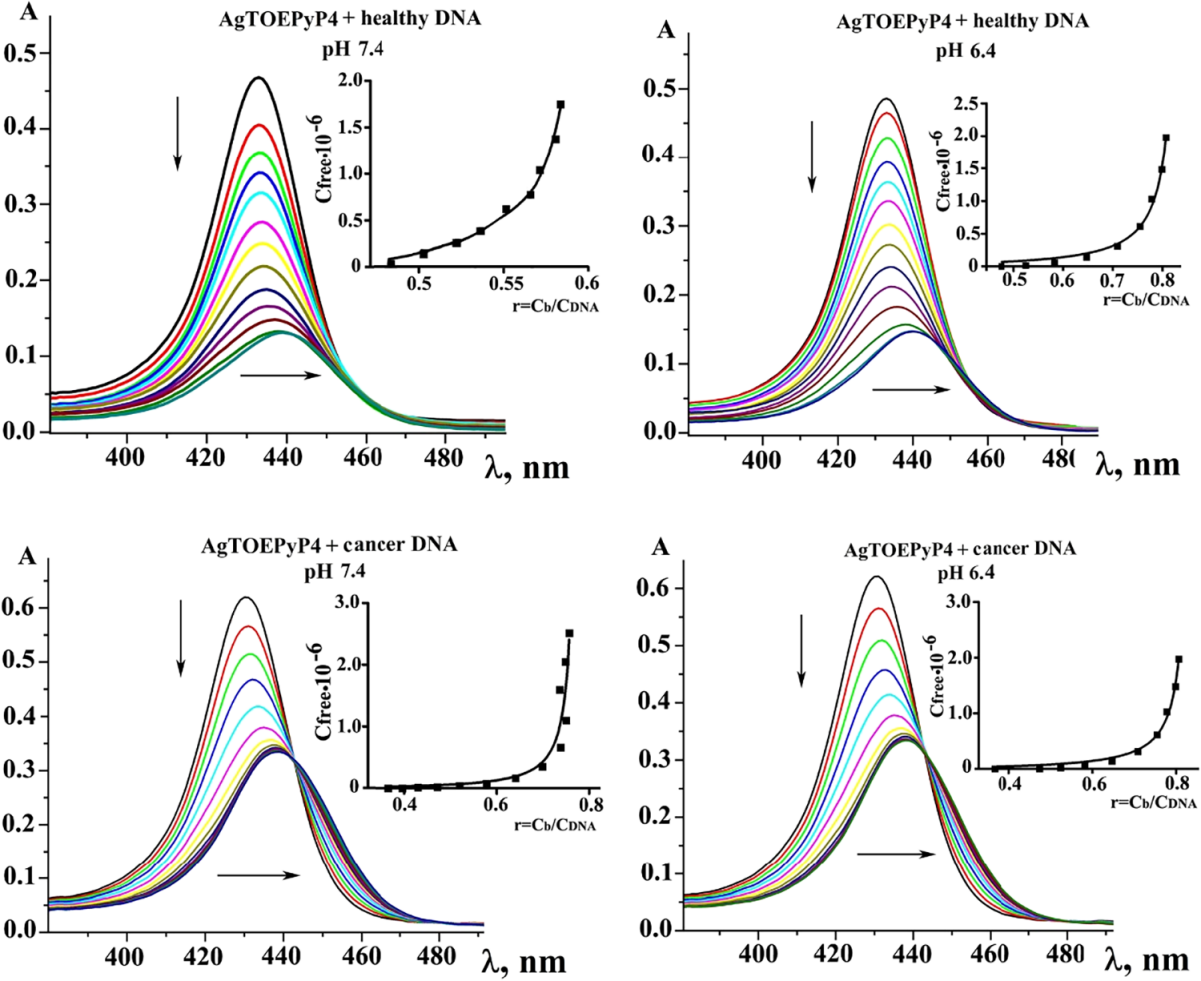
Absorption spectra and corresponding binding isotherms (insets) of AgTOEPyP4 in the Soret region obtained during titration with healthy and tumor DNA at pH 7.4 and pH 6.4. The insets show the results of fitting eq 3 to the experimental data processed according to eq 1.

Because even small changes in extracellular pH can influence tumor development and progression, we investigated porphyrin–DNA interactions at pH 7.4 and pH 6.4, representative of physiological and tumor-associated acidic environments, respectively.

As shown in Figures 4 and 5, both porphyrins exhibited similar spectral responses upon DNA binding, characterized by pronounced hypochromicity and a bathochromic (red) shift of the Soret band. Analysis of the titration data using eqs 1 and 3 enabled determination of the binding constants (*K*_b_) and exclusion parameters (*n*), which are summarized in Table 1. The fitted parameters were obtained with small standard errors, indicating an excellent agreement between the experimental data and the theoretical model. Representative fits of eq 3 to the processed experimental data are shown in Figure 6.

**Figure 6:**
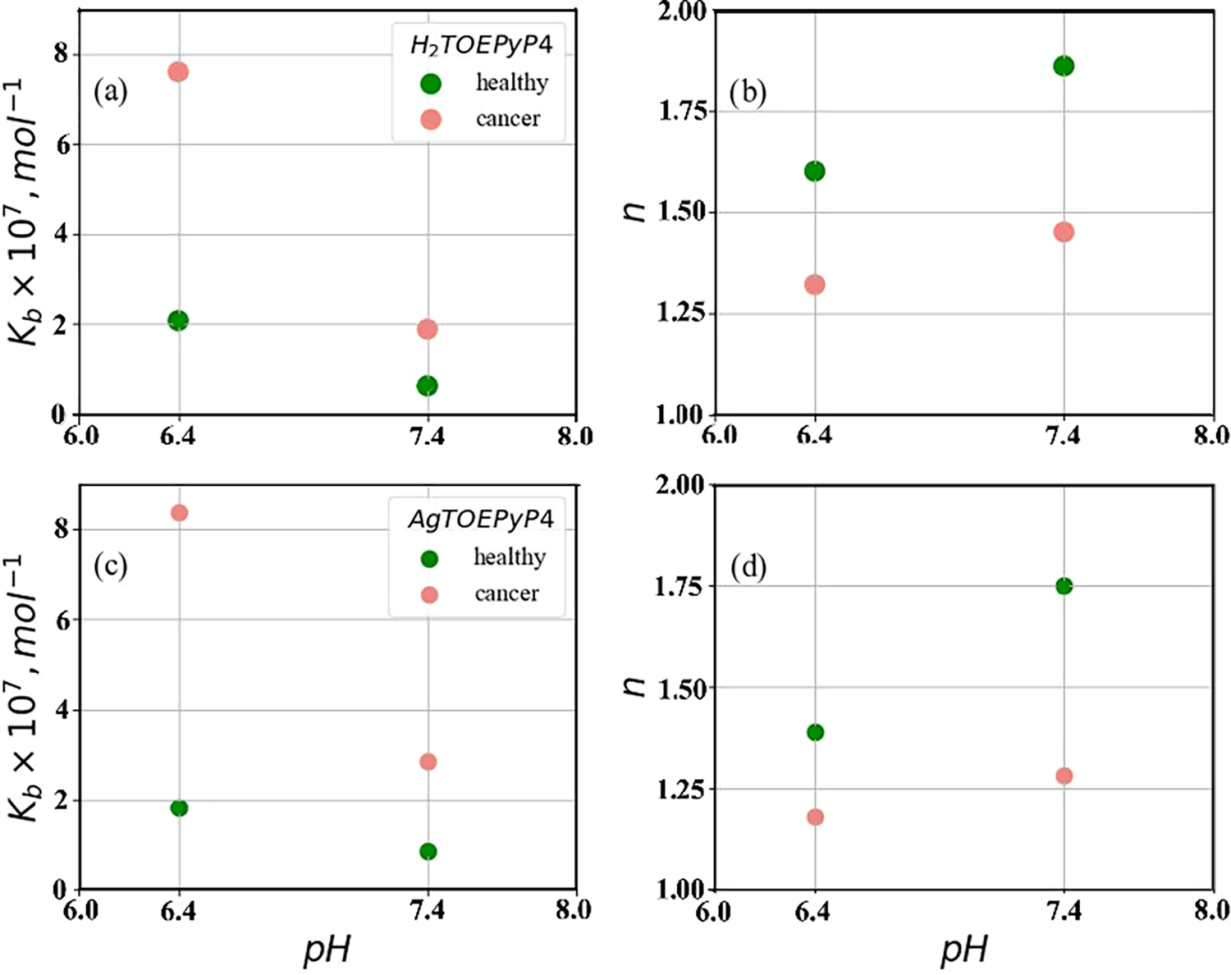
Binding parameters of H_2_TOEPyP4 and AgTOEPyP4 with healthy and tumor DNA at pH 7.4 and pH 6.4, obtained from fitting the absorption titration data shown in Figures 4 and 5. Panels (a, c) present the binding constants (*K*_b_) for H_2_TOEPyP4 and AgTOEPyP4, respectively, whereas panels (b, d) show the corresponding exclusion parameters (*n*). Green symbols represent healthy DNA, and pink symbols represent tumor DNA, as indicated in the legend. The errors associated with the obtained parameters are smaller than the symbol sizes (see Table 1).

**Table 1:** Binding Constants and Exclusion Parameters (*K*_b_, *n*) of Porphyrins H_2_TOEPyP4 and AgTOEPyP4 with Healthy and Cancer DNA at pH 7.4 and pH 6.4

complex	pH	*K*_b_ × 10^7^, M^–1^	*n*
H_2_TOEPyP4 + healthy DNA	7.4	0.63 ± 0.15	1.86 ± 0.10
	6.4	2.08 ± 0.10	1.60 ± 0.15
H_2_TOEPyP4 + cancer DNA	7.4	1.89 ± 0.20	1.45 ± 0.10
	6.4	7.61 ± 0.21	1.32 ± 0.15
AgTOEPyP4 + healthy DNA	7.4	0.84 ± 0.10	1.75 ± 0.15
	6.4	1.82 ± 0.15	1.39 ± 0.10
AgTOEPyP4 + cancer DNA	7.4	2.85 ± 0.20	1.28 ± 0.15
	6.4	8.37 ± 0.15	1.18 ± 0.11

For healthy DNA, the binding constants (*K*_b_) of H_2_TOEPyP4 and AgTOEPyP4 at pH 7.4 were 0.63 × 10^7^ and 0.84 × 10^7^ M^–1^, respectively. At pH 6.4, the corresponding binding constants increased to 2.08 × 10^7^ M^–1^ for H_2_TOEPyP4 and 1.82 × 10^7^ M^–1^ for AgTOEPyP4.

These results demonstrate that decreasing the pH by one unit substantially enhances the affinity of both porphyrins for the healthy DNA. Specifically, the binding constant increased approximately 3-fold for H_2_TOEPyP4 and approximately 2-fold for AgTOEPyP4 upon acidification. In contrast, the exclusion parameter (*n*) exhibited only a slight decrease at a lower pH for both porphyrins.

Thus, our investigation of the complex formation of H_2_TOEPyP4 and AgTOEPyP4 with tumor DNA under acidic conditions revealed a substantial increase in the binding affinity.

The binding constants of both porphyrins for tumor DNA in an acidic environment were approximately 1 order of magnitude higher than those observed for healthy DNA under physiological conditions. In contrast, the exclusion parameter (*n*) was lower for tumor DNA than for healthy DNA. This finding suggests that a greater number of porphyrin molecules can bind to tumor DNA, resulting in a binding density higher than that observed for healthy DNA.

## Discussion

Cellular metabolism, including redox reactions, the synthesis and degradation of proteins, lipids, and carbohydrates, enzyme activity, membrane permeability, and numerous other physiological processes, as well as the formation of porphyrin–DNA complexes highly dependent on pH and ionic strength.^24,25^

Our results show that the binding constants of both porphyrins increase significantly under acidic conditions (Figure 6a,c), which are characteristic of the tumor microenvironment. At the same time, the exclusion parameter (*n*) decreases (Figure 6b,d), indicating an increase in the number of available binding sites on the DNA molecule. One possible explanation is that DNA protonation at a lower pH reduces the shielding of negatively charged phosphate groups by Na^+^ ions. As a consequence, structural changes in the DNA helix may occur, including an increase in the distance between adjacent base pairs and partial unwinding of the double helix, thereby facilitating porphyrin binding.^26,27^

Three modes of porphyrin binding to DNA are generally recognized: intercalation, external self-stacking, and nonspecific external binding.^28^ The preferred binding mode depends on the structure of the porphyrin, including the nature of the central metal ion and the peripheral substituents. In AgTOEPyP4, the Ag­(II) ion is coordinated within the plane of the porphyrin macrocycle with a coordination number of four. Consequently, both H_2_TOEPyP4 and AgTOEPyP4 can interact with duplex DNA through intercalation at low porphyrin/DNA ratios and through external binding at higher ligand concentrations. As we recently demonstrated, the low porphyrin/DNA concentration range near the isosbestic point is associated with an intercalative binding mechanism and is accompanied by the appearance of negative induced circular dichroism signals.^29^ The positively charged silver ion located within the porphyrin plane may further enhance electrostatic interactions with DNA, contributing to the higher affinity of AgTOEPyP4 compared to that of the metal-free porphyrin.

Taken together, our *in vitro* findings demonstrate that the affinity of H_2_TOEPyP4 and AgTOEPyP4 for DNA increases under acidic conditions. This finding is consistent with the results reported by Zhao et al.,^13^ who observed stronger binding affinities under acidic conditions than under neutral conditions for a different cationic porphyrin interacting with both G-quadruplex and duplex DNA. Furthermore, tumor DNA differs from healthy DNA in nucleotide sequence, methylation status, secondary structure, and local charge distribution, all of which may influence the porphyrin binding. We therefore hypothesize that the enhanced affinity of our porphyrins for tumor DNA is associated with structural and physicochemical alterations characteristic of cancer-associated DNA. The lower exclusion parameter values observed for tumor DNA indicate more porphyrin binding sites. In addition, the acidic tumor microenvironment may contribute to the accumulation and retention of porphyrins in the tumor cells. These conclusions are consistent with our previous findings.^30^

Photodynamic therapy is an established anticancer strategy that employs porphyrins and light to generate reactive oxygen species and induce cell death.^31,32^ The combination of enhanced affinity for tumor DNA, preferential accumulation in acidic tumor environments, and the ability to generate singlet oxygen (^1^O_2_) and trigger photophysical and photochemical processes makes these porphyrins promising candidates for anticancer therapy.^22^

## Conclusion

We demonstrate that an acidic pH significantly enhances the binding affinity of H_2_TOEPyP4 and AgTOEPyP4 toward tumor DNA *in vitro*. The lower values of the exclusion parameter indicate that a greater number of porphyrin molecules can bind to tumor DNA than to those of healthy DNA. Because tumor tissues exhibit enhanced glycolytic metabolism and increased lactate production, their extracellular environment is more acidic than that of the normal tissues. The preferential binding of these porphyrins to tumor DNA under acidic conditions highlights their potential for the selective targeting of cancer cells and supports their further development as candidates for anticancer therapy.
